# The Backward Child of the Well-To-Do

**Published:** 1905-07-15

**Authors:** 


### THE BACKWARD CHILD OF THE WELL-TO-DO.

### A HOME SCHOOL.

On a beautiful Sunday afternoon in May we paid a visit to Fernhill Manor, at New Milton, over which Miss Mabel Anderson presides. The delightful old house conveyed a feeling of homelike cheerfulness and repose, and is surrounded by charming old-fashioned grounds. Most of the little ones were at a flower service when we arrived, but soon afterwards the little family returned full of the incidents of the day and eager to give an account of their experiences. The happy excitement which affected one and all almost banished the element of pity from our feelings. In the case of some of the children the abnormal was hardly noticeable, and the marked absence of self-consciousness or awkwardness would have been commendable in any group of children. Throughout our visit we were constantly encountering evidences of the careful moral, as well as mental education which is bestowed by Miss Anderson and her assistants upon the children. It was delightful to observe how the more capable showed themselves ever ready to help the less fortunate, and praise of one seemed to give pleasure to all the little family. We paid a visit to the schoolroom and admired the excellent results of patience on the part of the teachers and perseverance on that of the pupils. Those who have not studied the subject know little of the careful fundamental education necessary for the development of the "simple" minded child. The building up of body and mind simultaneously, with the object of restoring or producing some amount of co-ordination, is a science, the results of which are quite extraordinary and most creditable to those who undertake the work. Much knowledge, infinite patience, faith and even love are necessary. The absence of any one of these imperils success. How many unfortunate children, helpless in their affliction, are able to secure all this? We do not hesitate to say that within the ordinary home the backward child can never have the best chance of development, even if it is surrounded with loving care and the best of teachers. It must either be isolated or suffer from the thoughtless impatience of more gifted children. Few parents realise the bitter suffering endured, sometimes daily, by their unfortunate child, whose increasing sullenness or passionate temper and incapacity to learn they accept as an inevitable and often humiliating misfortune. The cruelty is unintentional, but it is none the less real. For many, however, it is avoidable, whilst such establishments as Fernhill Manor exist. If the whole aspect of the case were better understood we feel that there are few who would not even sacrifice something to give their child an opportunity of happiness and development. The improvement in the child's condition would be an ample reward. Under proper conditions the most hopeless cases become amenable to discipline, and obtain a self-control which is quite surprising. We observed a striking instance of this in the case of one nice little girl, who is an inmate of Fernhill Manor, who joined us at tea (her much-prized Sunday privilege) during our visit. Her manner was pleasant, she was most observant, and her remarks were quite intelligent and amusing. When we commented upon her polite attention to our wants, Miss Anderson remarked that when she arrived she resisted vigorously any request for small services, and exhibited most unamiable qualities. This child was of the type known as "Mongolian" in professional circles. The deficient of this type are often liable to heart and other troubles, besides needing great care to obtain the best results of mental development. They should therefore, when possible, always be placed under trained supervision.

Before leaving we watched the little ones at tea, and paid a visit to their pretty sleeping quarters, and to a poor baby, a victim to bronchitis, whose sufferings had deprived its kind guardian of more than one night's rest. Miss Anderson is a nurse of great experience; she has besides the highest qualifications for the task she has undertaken, having for some years superintended one of the most important establishmen ts for feeble-minded children. She is in fact an authority on the training and management of backward children, and is recommended by some of the highest medical authorities on the subject, amongst whom are Dr. Shuttleworth, Dr. Francis Warner, and Dr. David Ferrier. Miss Anderson is ably assisted. She has a kindergarten teacher and lady nurses, and a physician visits the home regularly. Her ter ms are very moderate. No one who visits Fernhill Manor can fa i to be struck by the success of Miss Anderson's methods, or the well-being and happiness of the children. Miss Anderson loves her work, and it prospers accordingly. Fernhill Manor is most healthily situated in charming country, not far from the sea, and close to Milton Station on the South-Western Railway.

